# Primary Cutaneous Aspergillosis in Immunocompetent Adults: Three Cases and a Review of the Literature

**DOI:** 10.7759/cureus.6600

**Published:** 2020-01-08

**Authors:** Vildan Avkan-Oğuz, Muammer Çelik, Ismail S Satoglu, Mahmut Cem Ergon, Ahmet emrah Açan

**Affiliations:** 1 Infectious Diseases and Clinical Microbiology, Dokuz Eylul University, School of Medicine, Izmir, TUR; 2 Orthopaedics and Traumatology, Dokuz Eylul University Faculty of Medicine, Izmir, TUR; 3 Medical Microbiology, Dokuz Eylul University, School of Medicine, İzmir, TUR; 4 Orthopaedics and Traumatology, Balıkesir University, Medical Faculty, Balıkesir, TUR

**Keywords:** aspergillosis, immunocompetent adults, case, treatment, management, fungal, voriconazole, infection

## Abstract

Primary cutaneous aspergillosis (PCA) can rarely affect immunocompetent people. There is limited knowledge about the prevalence, diagnosis and management of the disease because there are only case reports or small case series in the literature. For this reason, the diagnosis and treatment of three immunocompetent adult patients diagnosed with PCA were discussed by reviewing the literature. In the current report, in addition to treatment with voriconazole for 8-12 weeks we performed repeated surgical debridement for the treatment of these cases. After two negative tissue cultures, the wounds were either successfully closed primarily or reconstructed using a skin graft. Management of PCA cases will become easier as more reports and further studies of PCA contribute to our shared knowledge. Currently, the most appropriate management approach is to make individualized treatment decisions according to the patients’ clinical features and treatment response which includes several surgical debridement as well as antifungal therapy.

## Introduction

Skin and soft tissue infections caused by Aspergillus spp. (cutaneous aspergillosis) is an uncommon manifestation of aspergillosis. Cutaneous aspergillosis is defined as primary or secondary depending on the initial location of involvement and the etiological mechanisms [1]. Primary cutaneous aspergillosis (PCA) occurs at burns, catheter inlets and plaster adhesion regions that disrupt skin integrity, in areas of maceration or repeated skin trauma, in traumatic patients or in surgical wounds by direct inoculation. Secondary cutaneous aspergillosis (SCA) occurs by direct spread from adjacent focal points such as the lungs or paranasal sinus, or by hematogenous spread. SCA predominantly affects immunosuppressed patients such as those with hematological malignities who are stem cell recipients, and is more common compared to PCA [1,2]. PCA may disseminate and cause systemic infection, which can lead to longer hospitalization stays and increased treatment costs, as well as morbidity ending in limb loss and mortality [3,4].

The literature on primary cutaneous aspergillosis is limited to case reports and small case series; therefore, there is a paucity of data regarding the prevalence, diagnosis, and management of the disease [1]. The aim of this study was to discuss three immunocompetent adult patients diagnosed with PCA during follow-up in the orthopedics unit, two following trauma and one following fasciotomy, and to review the relevant literature. Informed consent to report individual cases were obtained from each patient.

## Case presentation

Case 1

**Figure 1:**
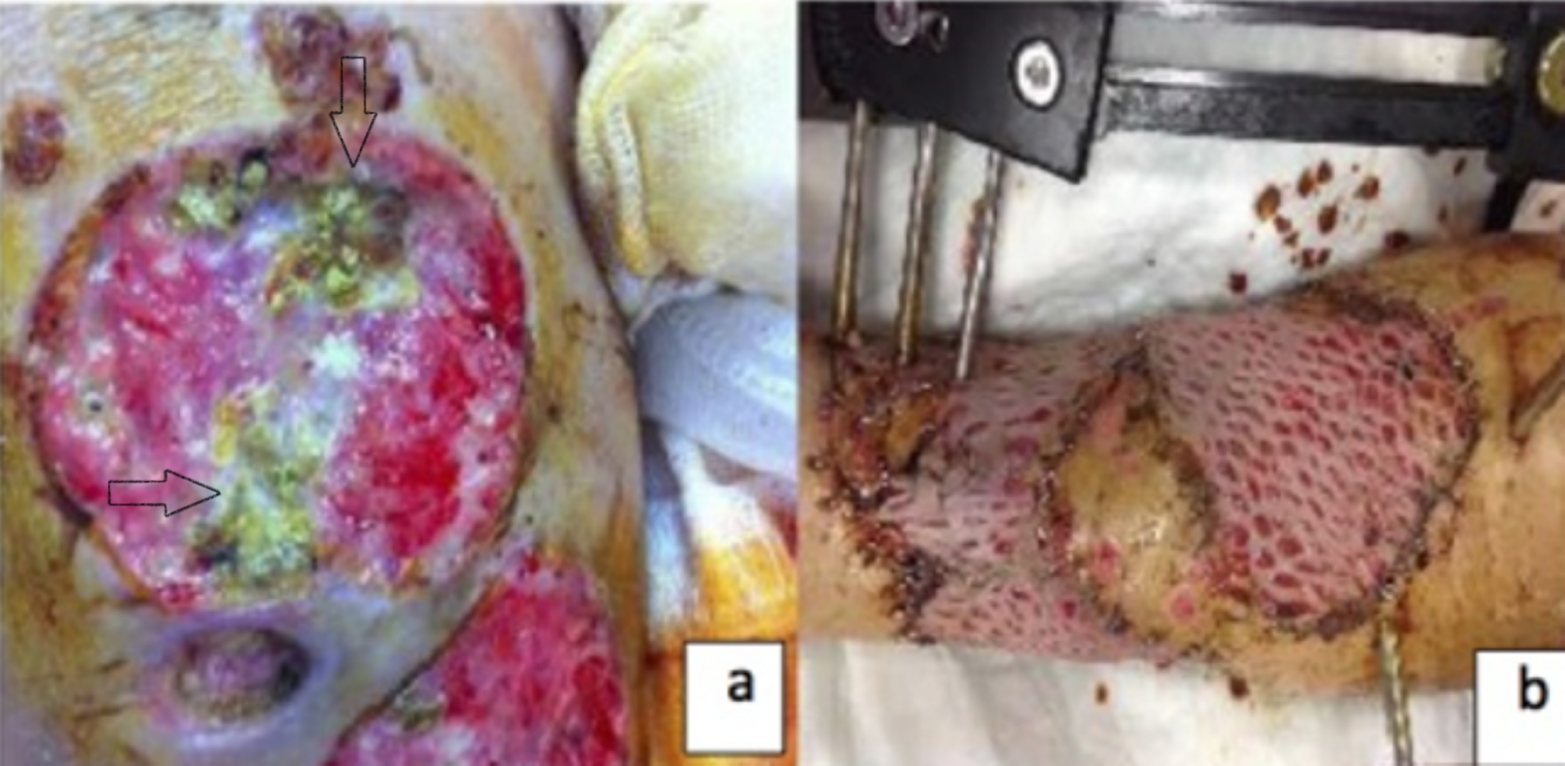
(a) Before treatment when the patient diagnosed with aspergillosis. (b) Following voriconazole and multiple debridement treatment, just after the granulation tissue was grafted.

A 53-year-old female patient presented with left tibia-fibula fracture and an anterior leg wound with exposed bone due to a motorcycle accident. Fracture treatment was combined with repeated surgical debridement of the wounds with large tissue defects. The patient’s wound dressing was changed regularly, and tissue culture was obtained during surgical debridement when necrotic tissue suggesting infection was observed in the wound. Aspergillus flavus was isolated from the tissue culture and obtained intraoperatively. Two additional tissue cultures were obtained in order to distinguish between colonization and infection. Fungal spores and hyphae branching at acute angles were observed in direct examination and mold growth was identified in the cultures. Because the infectious necrotic areas persisted despite surgical debridement and A. flavus growth was observed in three tissue cultures, antifungal treatment was initiated with intravenous voriconazole at a loading dose of 6 mg/kg twice daily and maintenance dose of 4 mg/kg twice daily. A superabsorbent wound dressing (Sorbact©, Abigo, Sweden) was used for daily dressing. This wound dressing reduces the microbial load in the wound by binding bacteria and fungi in a humid environment. No growth was observed in tissue cultures obtained in the third week of systemic antifungal therapy and treatment with superabsorbent wound dressing and repeated surgical debridement. After obtaining a negative culture result and while still receiving antifungal therapy, tissue repair was performed using a split thickness skin graft obtained from lateral right thigh. Voriconazole treatment was continued for 12 weeks (eight weeks intravenous + four weeks oral 200 mg twice daily). The patient’s pre- and post-treatment photographs are shown in Figure 1a, 1b.

Case 2

**Figure 2:**
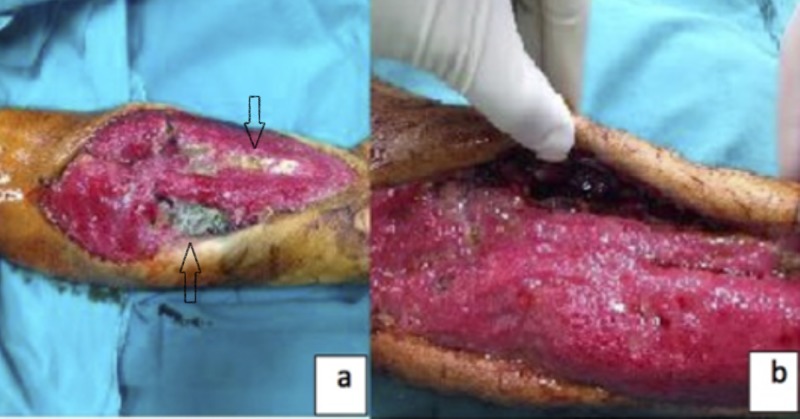
(a) Volar side of the patient’s forearm before treatment when the patient was diagnosed with aspergillosis. (b) Volar side of the patient’s forearm following voriconazole and multiple debridement treatment, just before the granulated wound was primarily closed.

A 39-year-old male patient presented to the emergency unit with burns and carbon monoxide poisoning. Areas of hyperemia consistent with first degree burn were identified in the right pectoral region, on the anteromedial right thigh, and the anterolateral right forearm. Fasciotomy was performed in the right forearm, right thigh, and right lower leg due to the development of compartment syndrome in the right arm and leg. The patient remained in the intensive care unit for 16 days for carbon monoxide poisoning and acute renal failure. While in the intensive care unit, he was taken to the operating room twice for debridement due to necrosis in the fasciotomy sites. Septate hyphae branching at acute angles was observed in direct examination and growth of A. flavus was identified in two tissue cultures. Treatment with intravenous voriconazole (loading dose of 6 mg/kg twice daily, maintenance dose of 4 mg/kg twice daily) and empirical antibiotic tigecycline (loading dose of 100 mg once daily, maintenance dose of 50 mg twice daily) was initiated. Vacuum-assisted closure (VAC) therapy was applied. No growth was observed in tissue cultures taken in the second week of antifungal therapy. After obtaining a negative tissue culture result, the wound was closed primarily. The patient’s general condition improved and his acute renal failure regressed. After 17 days of intravenous voriconazole, oral voriconazole 200 mg twice daily was administered as sequential therapy. The treatment lasted eight weeks in total. The patient’s pre- and post-treatment photographs are shown in Figure 2a, 2b.

Case 3

**Figure 3:**
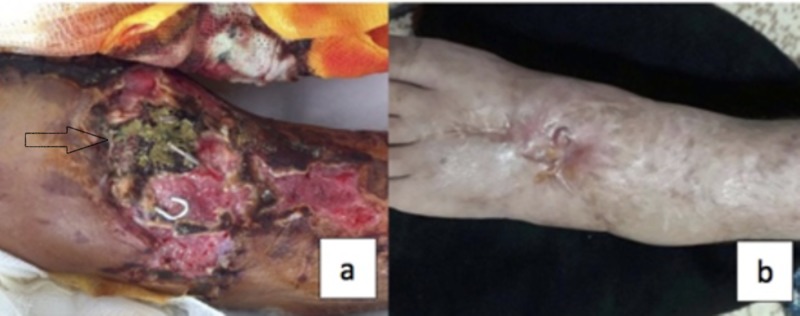
(a) Dorsum of the patient’s foot before treatment when the patient was diagnosed with aspergillosis. (b) Dorsum of the patient’s foot about one year after treatment, the patient has mild weakness of dorsiflexion of his ankle due to his extensor tendon repair.

A 59-year-old male patient presented after a motorcycle accident with fractures of the left distal radius, left fifth metacarpus, left tibial plateau, and left second and third metatarsi, as well as wounds on the anterior surface of the left tibia and the dorsal surface of the left foot. The patient underwent emergency surgery to repair the lacerated extensor tendons in his left foot and injured dorsalis pedis artery was ligated. He underwent another operation three days after admission to repair his left distal radius fracture and metatarsal fractures. In the third week, the left tibia plateau fracture was fixed with a hybrid Ilizarov ring and monolateral Schanz screws to tibial shaft and the necrotic tissue was debrided. Extensive necrotic lesions formed in the wounds on the dorsum of the patient’s left foot and his anterior tibia. Empirical treatment with tigecycline and ciprofloxacin was initiated based on a diagnosis of complicated skin/soft tissue infection. Tissue cultures were obtained from the wound on the fifth day of antibiotic therapy. Due to the persistence of extensive necrosis after nine days of antibiotherapy, tigecycline and ciprofloxacin were replaced with imipenem and teicoplanin. On the same day, wound debridement was performed in the operating room and tissue culture samples were obtained intraoperatively. A. flavus growth was observed in the initial tissue culture, and A. flavus and A. fumigatus growth was observed in the tissue cultures obtained intraoperatively. Based on the diagnosis of PCA, intravenous voriconazole at a loading dose of 6 mg/kg twice daily and maintenance dose of 4 mg/kg twice daily was added to the patient’s treatment. Debridement was repeated after 10 days and tissue cultures were obtained. Tissue culture was again positive for A. flavus. VAC treatment was started after a second debridement. No growth was observed in tissue cultures obtained on day 10 and day 17 of antifungal therapy. The wounds turned to healthy granulation tissue, and the patient underwent repair using grafts after completing four weeks of antifungal therapy. Intravenous voriconazole therapy was discontinued after 35 days and treatment with imipenem and teicoplanin was discontinued after 44 days, and the patient was discharged. The patient received oral voriconazole for three more weeks on an outpatient basis, after which treatment was discontinued. The patient’s pre- and post-treatment photographs are shown in Figure 3a, 3b.

## Discussion

Primary cutaneous aspergillosis is a rare infection and there is no definitive data regarding its prevalence. Patterson et al. reported cutaneous involvement in 5% and 4% of invasive aspergillosis cases, respectively [4,5]. In a more recent study conducted in France, it was reported that cutaneous involvement was found in 1% of 1,410 patients with confirmed or probable invasive aspergillosis diagnosed between 2005 and 2010 [6].

Although the infection is usually observed in immunocompromised patients, it may also occur less frequently in immunocompetent individuals. Tatara et al. reviewed 130 PCA cases reported in the literature between 1967 and 2015 [7]. They determined the underlying risk factor as hematologic malignancy in 28.5%, HIV/AIDS in 15.4%, being an infant in 12.3%, solid organ transplantation in 9.2%, burns in 6.2%, use of corticosteroids in 4.6%, stem cell transplantation in 3.8%, diabetes mellitus in 2.3% and trauma in 2.3%, but emphasized that no risk factor was found in 11.2% of the cases. We did not identify an underlying disease that may cause immune suppression in any of the three patients included in our study.

In order to review other published cases similar to those in our study, we searched the literature for primary cutaneous aspergillosis which yielded 125 articles published between 1967 and 2017 (searched in Pubmed, web of science, ulakbim). Articles published in either English or Turkish were selected, resulting in the elimination of 11 articles published in other languages. A total of 168 PCA cases were reported in 114 articles. The number of PCA cases in immunocompetent individuals was rather low in those articles. Nine cases of post-traumatic or post-operative PCA in immunocompetent individuals were compiled from eight articles in the literature and are presented in Table 1 [8-15]. One of these was a cutaneous aspergillosis case caused by A. terreus in a seven-year-old child reported from Turkey and another was a 70-year-old male patient with A. flavus and C. guilliermondii infection [11,12]. The three cases presented in our study comprise the first PCA series reported in immunocompetent adults in Turkey. Apart from these, the PCA cases previously reported from Turkey involved four immunosuppressed patients and one infant [16-19].

**Table 1:** Cases of posttraumatic or postoperative primary cutaneous aspergillosis in immunocompetent individuals published in the literature.

Author and year	Age, gender	Risk factors	Clinical presentation	Microscopy	Pathology	Treatment	Reference
Sharma et al., 2013	65, F	Farmer, trauma to the right foot one month earlier	Multiple erythematous nodules and plaques in the extremities and torso	Septate hyphae branching at acute angles	A. tamari	Itraconazole four weeks, no surgery	[8]
Tak et al., 2013	4, F	Traffic accident, large tissue defect in the right lower extremity	Green discharge following repeated surgeries and dressings for the wound	Hyaline septate hyphae	A. flavus and A. terreus	Voriconazole one week IV, two weeks oral	[9]
Martin et al., 2012	6, M	Traffic accident, pulmonary contusion and mediastinal hematoma, systemic steroid therapy	Erythematous bullous lesion with central necrosis on the proximal aspect of the left forearm	No data	A. fumigates	Voriconazole 21 days in, 10 days oral, no surgery	[10]
	2, F	Traffic accident, pulmonary contusion and thoracic spondylolisthesis, systemic steroid treatment	Multiple indurated erythematous papules with central eschar in the extremities	No data	A. fumigatus	Voriconazole six weeks, no surgery	
Türkşen et al., 2010	70, M	Trauma sustained while woodcutting in the garden	Painful necrotic wound in the middle finger of right hand for two months	Septate hypha branching at dichotomous angles and blooming yeast	A. flavus, C. guilliermondii	Itraconazole one week oral, surgical excision	[11]
Özer et al., 2009	7, M	Left leg crushed under tractor, operated for contaminated open tibial fracture	Large wound with green exudate in the anteromedial aspect of the left leg from below the knee to the malleolus	Fungal hyphae	A. terreus	Surgical debridement, daily dressing with potassium iodide and repair with graft, no medical treatment	[12]
Romano and Miracco, 2003	39, F	Farmer, local trauma sustained a few months earlier	Suppurative painless slow-growing nodule on the posterior aspect of the right ankle	Septate uniform hyphae branching at acute angles	A. fumigatus	Surgical excision, no medical treatment	[13]
Chakrabarti et al., 1998	28, M	Operated twice for ischiorectal abscess	A 10-cm wound in the gluteal area with foul-smelling necrosis	Branching septate hyphae	A. flavus	Itraconazole six weeks, wide debridement	[14]
Sawyer et al., 1992	79, M	Operated for duodenum perforation due to blunt abdominal trauma	Necrosis of the surgical site on postoperative day 6; Postoperative myocardial infarction, pneumonia, and sepsis	No data	A. flavus	IV liposomal amphotericin B initiated, debridement twice Postop exitus	[15]

In order to differentiate PCA from other fungal pathologies and etiologies during diagnosis, obtaining tissue samples by biopsy for culture and histopathological examination is recommended [2]. In the first two cases of the current report, A. flavus growth was observed in the tissue samples obtained from the wound and in the third case, growth of A. flavus and A. fumigatus was observed and fungal hypha structures were identified in direct examination. While the most common agent in the other forms of invasive aspergillosis is A. fumigatus (66%), A. flavus is found less frequently (14%) [4]. A. fumigatus is a more common agent particularly in invasive pulmonary aspergillosis due to its smaller conidia [20]. Because PCA infection develops by direct inoculation due to disruption of the skin’s integrity, differences in conidia sizes among types of Aspergillus is of no importance. Therefore, A. flavus is the predominant agent in PCA. In a study reviewing PCA cases, A. fumigatus (42.3%), A. flavus (35.1%) and A. niger (10.8%) were reported as the three most common agents [7].

Voriconazole is recommended as the first-line systemic antifungal therapy for medical treatment and surgical debridement is recommended to supplement antifungal therapy. Aspergillus species damage blood vessels, causing delayed angiogenesis and wound healing, which leads to the formation of necrosis in the wound. Although there is recent evidence that aspergillus downregulates angiogenesis in infected tissues, one important histological feature of invasive aspergillosis is ischemia/necrosis caused by angioinvasion and thrombosis of vessels. Therefore, systemic antifungal therapy has limited penetration into the wound. Surgical resection and debridement performed together with antifungal therapy have a synergistic effect by reducing the fungal burden on the wound, thus increasing treatment success rates. Systemic antifungal therapy accompanied by surgical intervention has been associated with lower mortality and recurrence rates [7]. Due to the limited number of studies about PCA and the fact that the existing literature consists only of case reports or small case series, there is no definitive information about the duration of antifungal therapy or when to plan surgery. While treating the first case in the current report, we performed several surgical debridement and used superabsorbent wound dressing (Sorbact®) in addition to treatment with voriconazole for 12 weeks. After two tissue cultures resulted in no growth, the wound was successfully reconstructed using a skin graft. In the second and third cases, voriconazole was administered for eight weeks accompanied by debridement and VAC treatment. In the second case, again following repeated surgical debridement and daily wound care we were able to primarily close the wound. The third case also necessitated several debridement and daily wound care and was successfully reconstructed using a skin graft after obtaining two negative tissue cultures. Factors that increase the likelihood of successful treatment include at least eight weeks of systemic antifungal treatment with multidisciplinary watchful clinical observation, effective surgical debridement, local wound care, and closing the wound after obtaining at least two negative culture results. After effective surgical debridement and systemic antifungal treatment, infection does not recur.

## Conclusions

In conclusion, we believe that the most appropriate management approach is to make individualized treatment decisions according to the patients’ clinical features and the treatment should include antifungal therapy and repeated surgical debridement as many as possible to obtain negative cultures for aspergillus species.
